# A Miniaturized Ligand Binding Assay for EGFR

**DOI:** 10.1155/2012/247059

**Published:** 2012-04-08

**Authors:** Jochen M. Schwenk, Oliver Poetz, Robert Zeillinger, Thomas O. Joos

**Affiliations:** ^1^NMI Natural and Medical Sciences Institute at the University of Tübingen, Markwiesenstraße 55, 72770 Reutlingen, Germany; ^2^Science for Life Laboratory Stockholm, School of Biotechnology, KTH Royal Institute of Technology, P.O. Box 1031, 171 21 Solna, Sweden; ^3^Molecular Oncology Group, Department of Obstetrics and Gynecology, Medical University of Vienna, Währinger Gürtel 18-20, 5Q, 1090 Vienna, Austria

## Abstract

In order to study receptor abundance and its function in solutions or in homogenates from clinical specimen, methods such as sandwich or radioimmunoassays are most commonly employed. For the determination of epidermal growth factor receptor (EGFR), we describe the development of a miniaturized bead-based ligand binding assay using its ligand EGF as immobilized capture reagent. This assay was used to analyze lysates from cell lines, and the ligand-bound EGFR was detected using an EGFR-specific antibody combined with a fluorescence-based reporter system. In a proof-of concept study with lysates from breast biopsies, the assay allowed to classify breast cancer samples in accordance to clinically the relevant EGFR cut-off level. The study suggests that such a ligand binding receptor assay could become an integral part of protein profiling procedures to provide additional information about receptor functionality in addition to its abundance.

## 1. Introduction

Since their introduction in the 1960s [1], immunoassays based on radiolabeled ligands or antibodies have become well-established technologies in research and clinical chemistry. Today, antibody-based immunoassays have been transferred and applied to various technological platforms such as multiplexed and miniaturized formats used in proteomic experiments [2, 3]. These multiplexed assay systems now allow analyzing hundreds of proteins in a single affinity-based experiment, but, thus far, radioimmunoassay-like measurements of ligand binding active receptors have not yet been transferred in a miniaturized format.

In the context of a detailed analysis of breast cancer, the quantification of active epithelial growth factor receptor (EGFR) in clinical specimen is a prominent example for the application of a radio-ligand binding assay. The investigation of receptor tyrosine kinases such as the EGFR is of interest, as overexpression of EGFR is correlated with poor prognosis [4], and the expression rates of EGFR and hormone receptors were strongly inverse [5]. EGFR itself is a transmembrane-spanning protein, with an extracellular ligand binding and a cytoplasmatic tyrosine kinase domain [6], and its main ligand EGF is a polypeptide consisting of 53 amino acids that binds to domain I and III of the extracellular part of the receptor. Upon binding of EGF to EGFR, the receptor undergoes conformational changes [7]. These lead to a downstream activation of the NF-*κ*B transcription factor pathway to induce antiapoptotic and proliferated genes, which eventually results in cell growth. EGFR plays a key role in the regulation of essential normal cellular processes and in the pathophysiology of hyperproliferated diseases such as many epithelial cancer types like breast, ovarian, lung, colorectal or prostate cancer [8]. EGFR is also known to be activated in a variety of tumors via the promotion of its gene c-erbB1, leading to an overexpression [9] by mutation or ligand binding [10]. EGFR expression may even serve as a decision maker for EGFR-targeted therapies [11], and, recently, the use of specific monoclonal antibodies directed against the phosphorylated and mutant form of EGFR (EGFRvIII) was suggested as a valid predictor of response to therapy with cetuximab, an anti-EGFR antibody [12].

 To measure the levels of EGFR in tissue samples, sandwich immunoassays can be used [13]. Complementary to these, radio-ligand binding assays (RLB) perform a more classical approach to measure quantities of functional EGFR by employing ^125^I-labeled EGF [14]. This receptor ligand binding assay takes advantage of the strong interaction between the ligand EGF and EGFR, which bind with an affinity of ≤12 nM [15].

 In the study presented here, we have developed a miniaturized solid-phase-based ligand binding assay as a possible alternative to assays involving radioactive tracers. Instead of using an EGFR specific antibody, we have employed the ligand EGF as a reagent to capture and profile functional EGFR present in the samples. This assay only requires small amounts of sample to determine relative EGFR level in lysates from cell culture material as well as from patient samples. The study reveals that bead-based ligand binding assay offers a complementary system to sandwich immunoassays and radioimmunoassays.

## 2. Methods and Material

### 2.1. Preparation of Cell Samples

Frozen pellets of the human breast cancer cells BT-20 (ATCC no. HTB-19) were solubilized on ice with 2% (v/v) NP-40 (Sigma) in a buffer containing 50 mM Tris (Sigma), 120 mM NaCl, 1 mM CaCl_2_, 1 mM MgCl_2_, and 2% (v/v) protease inhibitor (Sigma). The cells were sonicated 4 times for 5 s (Branson Sonifier, Korea) and centrifuged at 13,000 rpm for 5 min. The supernatant was collected for further studies.

### 2.2. Tissue Samples

Membrane fractions of 46 breast cancer samples with EGFR levels between 0 and 600 fmol EGFR per mg protein were included in the study (Supplementary Table 1 available online at doi:10.1155/2012/247059). The EGFR level was analyzed by a radio-ligand binding assay [14]. Seventeen samples had an EGFR level ≤10 fmol/mg, and 29 samples had EGFR levels of 11–246 fmol/mg. One sample had an EGFR concentration of 600 fmol/mg. All samples had been stored at −80°C for about 10 years.

### 2.3. Coupling of Beads

Biotinylated EGF (EGF-Biot, Molecular Probes) was coupled to avidin-coated beads (LumAvidin beads, Luminex Corporation). In each coupling reaction, 2.5 × 10^5^ beads were used. Firstly, beads were washed twice in block-and-storage buffer (BSB) containing 1% bovine serum albumin (Carl Roth) in phosphate-buffered saline (pH 7.4). Washing steps were performed as follows: beads were sedimented at 10,000 g for 2 min, supernatant was removed, and beads were resuspended with 250 *μ*L BSB, vortexed, and sonicated. EGF was coupled onto two differently color-coded bead sets at EGF-Biot concentrations of 1.2 *μ*M and 0.3 *μ*M over 30 min under permanent shaking in BSB buffer. Beads were washed twice with BSB and stored in BSB buffer containing 0.01% sodium azide (Merck) at 4°C in the dark before use.

### 2.4. Miniaturized Ligand Binding Assay

Assays were performed in a 96-well microtiter plate with a filter-membrane bottom (Millipore) in BSB under permanent shaking at 650 rpm and 23°C in the dark. In each well, 30 *μ*L containing EGF-Biot beads (1000 per set) and 30 *μ*L of sample were mixed and incubated for 2 h. The beads were washed with 3 × 50 *μ*L BSB buffer on a vacuum manifold (Millipore). An anti-EGFR antibody (0.3 *μ*g/mL, mAb11, LabVision) was selected, and 30 *μ*L were added to each well. After an incubation of more than 60 min, the beads were washed again as above. A secondary R-phycoerythrin-labeled antibody (2.0 *μ*g/mL, goat anti-mouse, Dianova) with a volume of 30 *μ*L/well was incubated for 45 min. Finally, the beads were washed, and a final volume of 75 *μ*L BSB was added to each well.

For competition experiments, soluble EGF (sEGF, Biomol) was mixed with the sample and coincubated with immobilized EGF-Biot-coupled beads. For detection, anti-EGFR antibody mAb11 was applied at 1.25 *μ*g/mL and the labeled anti-mouse antibody at 5.0 *μ*g/mL.

### 2.5. Readout and Data Analysis

A Luminex LX100 system (Luminex Corporation) was used to determine the bead-bound reporter fluorescence. For each well, at least 100 events per bead ID were counted and the median fluorescence intensity (MFI) of the reporter dye was collected. Data was processed using R, a language and environment for statistical computing and graphics [16]. For the comparison of two groups with differing EGFR expression values, a *P* value was calculated with Student's *t*-test.

## 3. Results

**Figure 1:**
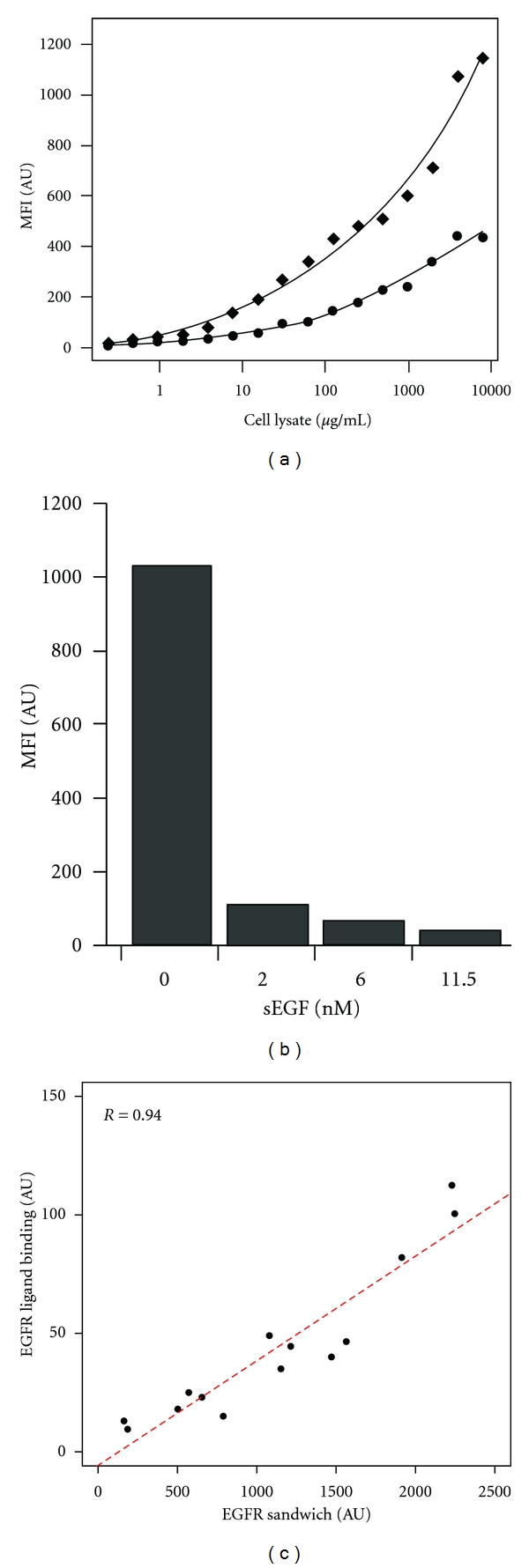
(a) Detection of EGFR in cell extracts. Beads coated with different concentrations of EGF were used to capture EGFR from a cell lysate in a concentration-dependent manner. The ligand EGF was immobilized at 1.2 *μ*M (diamonds) and 0.3 *μ*M (circles) on different bead types. Both bead sets were mixed and incubated with different amounts of BT-20 cell lysates. Captured EGFR was detected using an anti-EGFR antibody and a labeled secondary antibody. (b) Specificity of capture activity. A BT-20 cell lysate (500 *μ*g/mL) was coincubated with various concentrations of purified and unbiotinylated sEGF and beads coated with EGF-Biot (1.2 *μ*M). The amount of captured EGF receptor was strongly reduced at the presence of sEGF. This indicates that the bead-bound EGF-EGFR complex is captured in a specific fashion by applying an anti-EGFR antibody followed by a labeled secondary antibody. (c) Correlation of ligand binding and sandwich immunoassay. A series of tissue lysates with known EGFR concentrations were analyzed with both, a ligand binding assay and an immunoassay using an antibody as capture reagent. In both assays, the same anti-EGFR detection molecule was used and the profiles obtained reveal a correlation of 0.94, which indicated a good concordance between the two tests.

 In a first set of experiments, two concentrations of biotinylated EGF were immobilized onto beads, mixed, and applied to an assay with different amounts of total protein from cell lysates. For this study, BT-20 cells, known to express EGFR at level of 400 fmol/mg or 1.5 × 10^6^ copies per cell [17], were chosen. For the subsequent detection of bead-bound EGFR, an anti-EGFR antibody was chosen that did not interfere with the EGF-EGFR interaction, according to the data sheet. No cross-reactivity between immobilized EGF-biotin, the anti-EGFR antibody, and the labeled anti-mouse detection antibody could be detected (data not shown). In Figure 1(a), it is shown that both the amount of lysate employed as well as the ligand density on the bead surface influenced the measured signal intensity and no saturation effects were observed for the tested lysate protein concentrations. EGF coupled to the beads at a concentration of 1.2 *μ*M showed increased signal intensities compared to the bead ID coupled with a 4x lower ligand concentration, in addition to a broader intensity range for the 1.2 *μ*M loaded EGF beads. As the utilized BT-20 cells are known to express higher levels of EGFR, a sample protein concentration of 500 *μ*g/mL, reflecting 15 *μ*g of total protein per well, was chosen for the following studies to also facilitate the detection of EGFR in specimens with lower expression levels. To validate the specificity of the assay, meaning that EGF is capturing EGFR, BT-20 cell lysates were coincubated with nonbiotinylated soluble EGF (sEGF). In this competition assay, sEGF will bind to free EGFR binding sites and occupy them so that the immobilized EGF cannot bind to such a EGFR molecule. The results from this test (Figure 1(b)) showed a strong reduction in signal intensity at sEGF levels of 11.5 nM (96% reduction) and 2 nM (89% reduction). This clearly indicated that the observed interactions are due to EGFR-EGF binding. Moreover, we also compared the ligand binding assay to a sandwich immunoassay with both tests being performed on beads and utilizing the same detection system. As shown in Figure 1(c), a concordance of *r* = 0.94 was achieved for the profiles generated with breast cancer tissue samples (see below). This demonstrated that a protocol for a ligand binding assay was developed and that is allowed to provide specific and functional information for profiling the cell surface receptor EGFR in lysates.

**Figure 2:**
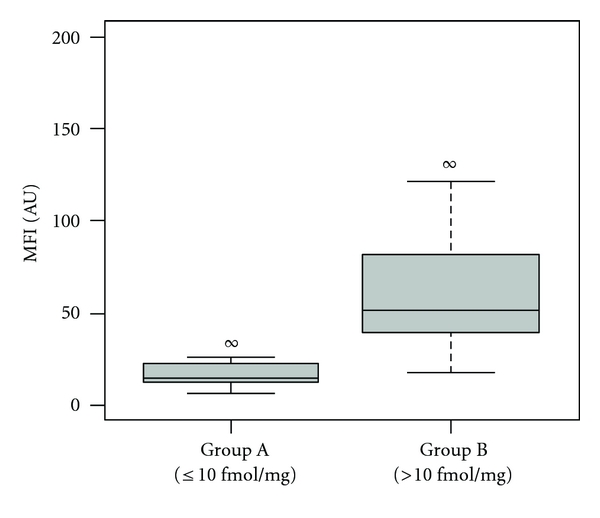
Analysis of breast cancer tissue samples. Forty-six breast cancer tissue samples originally analyzed for EGFR expression by a radio-ligand binding assay were reanalyzed in a bead-based ligand binding assay. A cut-off value of 10 fmol EGFR per mg protein, determined by radio-ligand binding assay, was used to divide the samples into group A (*n* = 17) with EGFR values ≤10 fmol/mg and group B (*n* = 29) with EGFR values >10 fmol/mg. Samples were measured in a random order, and a *P* value of 2.6e-11 was calculated between the two groups.

 Next, the EGFR ligand binding assay was applied in a proof-of-concept study to profile EGFR in 46 tumor samples derived from breast cancer patients. The tissue lysates were measured in random order at a total protein concentration of 0.5 mg/mL with the established protocol. The analyzed samples were grouped based on a clinically relevant cut-off level of 10 fmol/mg [5], which had been previously determined in radio-ligand binding experiments. As shown in Figure 2, 15 of 17 samples (88%) from group A with EGFR ≤10 fmol/mg were measured with MFIs ≤25 AU, while samples with MFI ≥50 AU were not affiliated to this group. For samples in group B with EGFR >10 fmol/mg, 52% had MFI values ≥50 AU, and, for the remaining 48% with MFIs ≤50 AU, the highest EGFR value was 42 fmol/mg. A sample containing 600 fmol/mg EGFR (not shown in Figure 2) was measured with an MFI >1000 AU thus served as a control. Between group A and B, a *P* value of 2.6e-11 was calculated and demonstrated that the ligand binding assay performed well to provide complementary evidence for separating samples with high and low EGFR values.

## 4. Discussion and Conclusion

Miniaturized ligand binding assays, as described here for EGF and EGFR, offer a radiation-free tool to profile and study target molecules via their in vivo interaction partners compared to conventional radioimmunoassays or other antibody-based methods. In the presented approach, we investigate the potential of the immobilized receptor ligand EGF to capture EGFR in a miniaturized bead-based assay format in which EGF was immobilized to avidin-coated beads via an N-terminal biotin modification. The chosen immobilization strategy enabled to maintain the binding properties of EGF and to achieve high signal intensities. Both, the EGF coupling concentration as well as the amount of the applied sample affected signal intensities. As described elsewhere, the extracellular domain of EGFR undergoes a conformational change upon ligand binding on the cell surface [7] and it seemed likely that such changes should occur even when EGFR is being bound by an immobilized capture ligand. Even though EGF-EGFR interactions take place with a free EGF binding to anchored or soluble EGFR in vivo, attaching EGF to a solid support did not hinder the two proteins from binding to each other.

In competition assays, it was revealed that the binding by detected immobilized EGF was affected by purified and soluble sEGF, thus confirming that the measured interactions were from EGF and EGFR. The strong inhibitory effect of soluble EGF is likely to reflect advantages in conformation adaptation of EGFR when binding in solution. Compared with purely antibody-based sandwich immunoassays, that were described earlier [18], the influence of conformational may have a greater influence on the ligand binding assay. This could be interpreted from the decreased intensity level that was found in ligand binding assays. In addition, this observation may eventually be a consequence of the fact that the total number of receptors accessible to ligand binding is lower due to the applied extraction procedure and sample storage, which may not keep all receptors in nondenatured and functional states.

To investigate the possible potential applicability of this approach in future clinical sample analysis, EGFR-characterized tumor samples from breast cancer patients were studied. The analyzed samples were grouped based on a clinically relevant cut-off level for EGFR, and it was possible to discriminate samples with expression levels below or above cutoff using the described assay setup. Even though this small proof-of-concept study may suggest that such miniaturized ligand binding approaches could have the potential to be a supplementary alternative to radioactivity-based tests, further testing and assay evaluations need to be performed.

The demonstrated miniaturization of receptor ligand binding assays allowed an analysis of potentially functional receptors from minimal sample amounts and would decrease costs by reducing the amount of material and reagents needed. The reduction of sample volume would be of particular importance for applications where only minimal amounts of specimen are available, such as the analysis of multiple tumor markers from biopsies. In addition, the miniaturized ligand binding assays may have the capability to supplement existing single- or multiplexed sandwich immunoassays to further increase the evidence for specific target presence and to add informatory value.

In conclusion, the ligand binding assay system demonstrates that ligand receptor-binding assays can be miniaturized and offer to become part of the variety of protein microarray-based approaches performed today. With new proteins and pathways emerging as indicators of disease, the procedure presented here describes a possibility to profile the interactions of proteins, such as cell surface receptors, in a miniaturized assay. It is possible that such assays are of value for variety of assays in the fields of proteomics and diagnostics.

## Supplementary Material

The supplementary table lists the samples from breast tissue obtained through biopsies. Forty-seven breast cancer tissue samples were available with EGFR values previously determined in radio-ligand binding assays.

## Abbreviations

EGFR: Epidermal growth factor receptor
EGR: Epidermal growth factor
EGF-Biot: Biotinylated EGF
sEGF: Soluble EGF
MFI: Median fluorescence intensity.
