# Supplementary material for: A Miniaturized Ligand Binding Assay for EGFR
Source: Int J Proteomics. 2012 Apr 8;2012:247059. doi: 10.1155/2012/247059 (PMC3332193; doi:10.1155/2012/247059)
Supplement: Supplementary file 1 — The supplementary table lists the samples from breast tissue obtained through biopsies. Forty-seven breast cancer tissue samples were available with EGFR values previously determined in radio-ligand binding assays. [file 247059.f1.pdf]

Supplementary Table 1

List of breast cancer tissue samples – Forty-seven breast cancer tissue samples were available together with their EGFR values previously determined in RIA assays.

Sample ID	EGFR [fmol/mg]	Sample ID	EGFR [fmol/mg]
S-01	42	S-25	1
S-02	38	S-26	72
S-03	46	S-27	9
S-04	6	S-28	246
S-05	12	S-29	6
S-06	8	S-30	10
S-07	115	S-31	16
S-08	46	S-32	31
S-09	24	S-33	135
S-10	22	S-34	4
S-11	82	S-35	0
S-12	11	S-36	2
S-13	8	S-37	12
S-14	21	S-38	3
S-15	7	S-39	600
S-16	16	S-40	33
S-17	10	S-41	66
S-18	14	S-42	14
S-19	122	S-43	13
S-20	42	S-44	0
S-21	11	S-45	4
S-22	13	S-46	25
S-23	18	S-47	5
S-24	5		
